# The role of Endocan as a Prognostic Biomarker in community-acquired pneumonia

**DOI:** 10.12669/pjms.35.1.280

**Published:** 2019

**Authors:** Senay Gunaydin, Mehmet Kucuk, Ulug Mutlu Gunaydin

**Affiliations:** 1 Dr. Senay Gunaydin, Department of Internal Medicine, Saglik Bilimleri University Okmeydani Education and Research Hospital, Okmeydani-Istanbul, Turkey; 2 Dr. Mehmet Kucuk, Associate Professor, Department of Internal Medicine, Saglik Bilimleri University Okmeydani Education and Research Hospital, Okmeydani-Istanbul, Turkey; 3 Dr. Ulug Mutlu Gunaydin, Department of Internal Medicine, Istanbul Medeniyet University, Unalan Street E-5-34700 Uskudar –Istanbul, Turkey

**Keywords:** Endocan, Patients, Pneumonia

## Abstract

**Objective:**

The aim of this study was to determine the importance of using endocan as a biomarker in deciding the setting of treatment and predicting prognosis in patients with community-acquired pneumonia (CAP).

**Methods:**

This prospective, case-control study was conducted at Okmeydani Training and Research Hospital between November 20, 2016 to March 20^th^ 2017. Blood samples were obtained from 63 patients who were admitted to internal medicine clinic due to CAP and 25 volunteers without active infection. Serum samples were centrifuged at 1000G for 15 minutes and stored at -20ºC. Samples were analyzed using human ESM1 (endocan) (Lot No: AK0017MAR0830) (Elabscience, Texas, USA) kit with Robonik (Mumbai, India) ELISA Plate Reader and Washer. Demographic and clinical data of the patients were recorded. CURB-65, qSOFA and Pneumonia Severity Index (PSI) scores were calculated. Primary endpoint of the study was 30-days mortality.

**Results:**

Mean serum endocan levels of the study group and the control group were 30.99±3.3 pg/ml and 246.5±49.95pg/ml, respectively. The difference between groups was statistically significant (p<0.005). 30-days mortality rate was 12.7% with eight patients, three of which died subsequently in the ICU. When patients were classified according to PSI and CURB-65 scores, endocan levels of PSI class ≥4 and CURB-65 ≥2 individuals were found to be significantly different than the control group. ROC analysis showed that serum endocan levels less than 64.96pg/ml has 85.2% sensitivity and 83.3% specificity for PSI class ≥4 and 82.4% sensitivity and 55.6% specificity for CURB-65 score ≥2.

**Conclusion:**

Serum endocan levels are significantly lower in patients with community-acquired pneumonia than the control group.

## INTRODUCTION

Community-acquired pneumonia (CAP) is the acute infection of the pulmonary parenchyma occurring in daily community life with simultaneous clinical and radiological consolidation in one or more lobes of the lungs. Pneumonia is the leading cause of infection-related deaths and is a major health problem because of its high mortality and morbidity. The causative microorganism cannot be determined in almost half of the cases with pneumonia. Thus, empirical antibiotherapy is necessary for the first-line treatment. CURB-65^1^ and Pneumonia Severity Index (PSI)^2^ scoring systems have been defined in order to facilitate the choice of accurate empirical antibiotics and the decision of inpatient vs. outpatient treatment. According to these systems, patients with CURB-65 score <2 and PSI score I, II and III can be treated in outpatient basis while others require inpatient management. Scoring systems prevent unnecessary hospital admissions and assure careful choice of empirical antibiotherapy according to the risk of mortality.

Acute phase reactants are used as biomarkers in determining the severity of pulmonary parenchyma infections. C-reactive protein (CRP) and procalcitonin (PCT) are among the most frequently used biomarkers in clinical practice.^3^

Endocan is a proteoglycan biomarker which is secreted from renal and pulmonary endothelial cells and can be detected in plasma.^4^ Previous studies have indicated that this proteoglycan can be used as a prognostic marker in endothelial dysfunction and sepsis.^5^,^6^ In this study, we investigated the correlation of endocan with CURB-65 and PSI scores used in pneumonia and whether it can be used as a prognostic factor.

## METHODS

Sixty three patients who were admitted to the internal medicine clinic in Okmeydani Training and Research Hospital between 11.20.2016 and 03.20.2017 with community-acquired pneumonia and 25 healthy volunteers with no active infection were included in the study. *Inclusion criteria* were age ≥18 years, patients with a diagnosis of community-acquired pneumonia and patients giving consent or volunteers. Pregnant patients and patients who did not give consent were excluded from the study.

### Research Questions

1. Are there any correlation of Endocan with CURB-65 and PSI scores used in pneumonia?
2. Can we use Endocan levels as a prognostic factor in pnomonia?

Serum samples of patients, who were admitted with the preliminary diagnosis of community-acquired pneumonia, were obtained within the first 24 hours of admission in order to measure serum endocan levels. Blood samples were centrifuges at 1000G for 15 minutes. The supernatant was transferred to an Eppendorf tube and was stored at -20ºC until measurement. Stored samples were analyzed using human ESM1 (endocan) (Lot No: AK0017MAR0830) (Elabscience, Texas, USA) kit with Robonik (Mumbai, India) ELISA Plate Reader and Washer.

Patients were evaluated on the first day of admission. CURB-65, PSI and qSOFA scores were calculated using the laboratory tests performed on the time of presentation (glucose, BUN, creatinine, Na, K, Cl, AST, ALT, LDH, GGT, bilirubin, arterial blood gas etc.) and patient history.

The correlations between endocan levels and the demographical characteristics of the patients with pneumonia, their biochemical profiles, CRP levels and CURB-65, PSI and qSOFA scores were evaluated.

Endocan levels were compared with PSI scores, CURB-65 scores and, if present, CRP and procalcitonin levels. The role of endocan as a biomarker in deciding if the patient requires admission to hospital and predicting patient prognosis was assessed.

The outcomes of the patients in pneumonia group (transferred to ICU, discharged, death etc.) were noted from patient files. For 30-days mortality rates, mortality data of the patients who were transferred to ICU were obtained with telephone. As for control group, blood samples were taken from 25 volunteers who presented to internal medicine outpatient clinics and whose physical examination revealed no sign of infection. Blood samples of the control group were centrifuged and stored the same way as the patient group.

### Statistical Analysis

The data analyses were performed using IBM SPSS Statistics Version 23 (Chicago, IL, ABD) and GraphPad Prism v.6.01 (GraphPad Software, Inc., San Diego, CA, ABD) software.

Normal distribution of numeric variables was evaluated with Shapiro-Wilk test, skewness and kurtosis. Normally-distributed numeric variables were expressed as mean and standard variation while non-normally distributed variables were expressed as median. The correlation between two numeric variables with normal distribution and a linear correlation was analyzed using Pearson’s test. The correlation between two ordinal variables without normal distribution and without linear correlation was analyzed using Pearson’s test. When comparing groups with normal distribution, independent samples t-test was used for situations with two groups while One-way ANOVA was used for situations with three or more groups. When comparing groups without normal distribution Mann-Whitney U-test was used for situations with two groups while Kruskal-Wallis H test was used for situations with three or more groups.

ROC (Receiver Operating Characteristics) analyses were used to determine the most accurate cutoff point for endocan and define sensitivity and specificity values. p<0.05 was considered statistically significant.

## RESULTS

**Fig.1:**
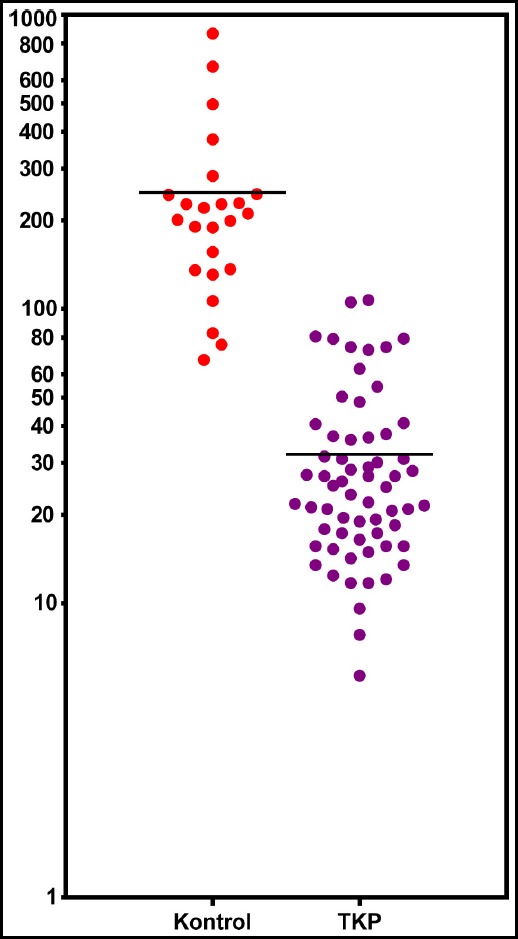
The distribution of serum endocan levels (pg/ml) in control and CAP patients (y axis is logarithmic)

Out of 63 patients with CAP included in our study, 36 (57.1%) were male and 27 (42.9%) were female. The sex distribution of the patients is shown in Fig.1. The mean age of the patients was 72.05±13.2 (range: 25-91). The mean age of males was 70.05±13.3, while the mean age of females was 74.11±13.1. The distribution of patients according to age was as follows: one patient in 3^rd^ decade, one patient in 4^th^ decade, one patient in 5^th^ decade, 6 patients in 6^th^ decade, 15 patients in 7^th^ decade, 17 patients in 8^th^ decade, 20 patients in 9^th^ decade and two patients in 10^th^ decade.

The mean age of 25 volunteers without active infection that were included in the study as the control group was 57.12±15.45. 36% of these patients were male and 64% were female. The distribution of the comorbid diseases of patients with community-acquired pneumonia is shown in Table-I.

**Table-I:** The Distribution of comorbidities.

Comorbid Disease	Number (n)	Percentage (%)
Hypertension	37	57.7
Diabetes Mellitus	27	42.8
Congestive heart failure	27	42.8
Chronic obstructive pulmonary disease	25	39.6
Chronic kidney disease	13	20.6
Solid tumor	13	20.6
Cerebrovascular disease	12	19.0
Dementia	11	17.4
Asthma	3	4.7

Thirty five (55.6%) of the patients had been using tobacco. six (9.52%) of the smoker patients were female and 29 (46.03%) were male. The mean duration of hospital stay was eight days (1-29 days). 49 patients (77.8%) were discharged while five patients (7.9%) were transferred to intensive care unit due to respiratory failure. five patients (7.9%) died and 4 patients (6.3%) refused treatment. 30-days mortality rate was 12.7% with eight patients.

Mean serum endocan level was 30.99±3.3 pg/ml in the pneumonia group while it was 246.5±49.9 pg/ml in the control group. Independent variables of the CAP group and the control group were evaluated with t-test. The difference between the group means was statistically significant (p<0.005). The distribution of serum endocan levels in control and CAP patients is shown in Fig.1.

The laboratory profile according to PSI classes of the patients with CAP is seen in Table-II. There was no statistically significant difference between PSI groups of the study patients in terms of white blood cell count and CRP levels. However, serum endocan levels were significantly different between PSI classes (p=0.04).

**Table-II:** White blood cell count, CRP and Endocan levels according to PSI class

	PSI class 2	PSI class 3	PSI class 4	PSI class 5	
White blood cell count	12565±3787	21833±9597	15034±6398	13410±7885	p=0.09
CRP	124.9±146	137.7±128.5	188.3±124.3	124.2±85	p=0.19
Endocan	125.5±198.3	25.9±9.8	22.5±8.8	54.3±69.1	p=0.04

The laboratory profile according to CURB-65 scores of the patients with CAP is seen in Table-III. There was no statistically significant difference between CURB-65 scores of the study patients in terms of endocan and CRP levels. However, white blood cell count was significantly different between groups of different CURB-65 scores (p<0.005).

**Table-III:** White blood cell count, CRP and Endocan levels according to CURB-65 scores

	CURB-65 0 point	CURB-65 1 point	CURB-65 2 points	CURB-65 3 points	CURB-65 4 points	CURB 5 points	
White blood cell count	23400±11570	18643±6574	12950±4961	13037±5446	11866±7029	44700	p<0.005
CRP	88.1±55.3	208.3±153.8	151.7±108.7	154.7±94.4	97.5±93.9	76	p=0.28
Endocan	28.1±8.35	22.2±5.8	46.1±76.3	48.6±66.8	60.7±85.4	19.5	p=0.86

**Fig.2:**
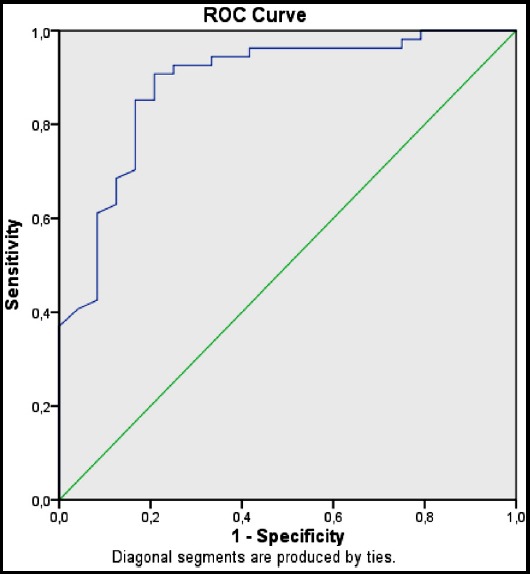
ROC curve comparing endocan levels with PSI class

ROC analysis was performed to evaluate the power of serum endocan levels at the time of hospital admission to predict PSI class ≥4. According to this analysis, endocan level is a good parameter to predict PSI class (AUC:0.88 and %95 CI 0.80-0.96). When cut-off value for endocan is considered to be 64.96 pg/ml, the sensitivity is 85.2% and the specificity is 83.3%, as shown in Fig.2.

**Fig.3:**
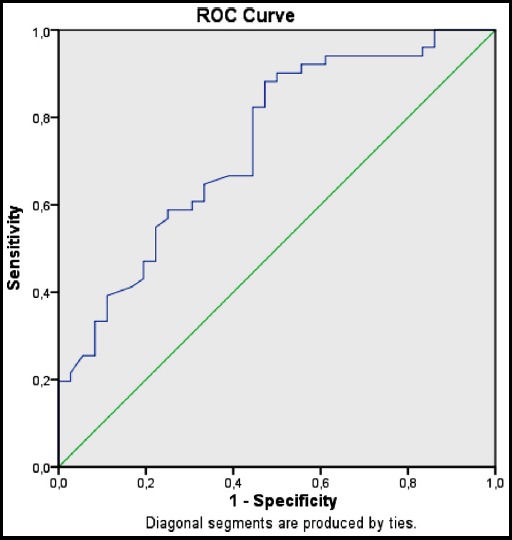
ROC curve comparing endocan levels with CURB-65 score.

ROC analysis was performed to evaluate the power of serum endocan levels at the time of hospital admission to predict CURB-65 score ≥2. According to this analysis, endocan level is a good parameter to predict CURB-65 score ≥2 (AUC: 0.73 and %95 CI 0.63-0.84). When cut-off value for endocan is considered to be 64.96 pg/ml, the sensitivity is 82.4% and the specificity is 55.6%, as shown in Fig.3. Endocan levels of all patients who died in our study were below 64.96pg/ml. The mean endocan level of passed-away patients was 19.04±8.8 pg/ml.

## DISCUSSION

Community-acquired pneumonia is a leading cause of hospitalization and mortality worldwide and brings a significant burden of treatment costs. Despite widespread use of broad-spectrum antibiotics and vaccination^7^, CAP is still the most common cause of infection-related deaths and the third leading cause of death overall. According to 2015 data, more than 3.2 million people in the world die because of pneumonia.^8^

When a physician encounters community-acquired pneumonia, it is critical to predict its prognosis and evaluate the treatment setting, whether it can be treated as outpatient or requires hospitalization or even intensive care unit treatment. Scoring systems, including CURB-65 and Pneumonia Severity Index, have been developed to ensure more objective patient selection. These scoring systems consider 30-days mortality rates. ≥2 points in CURB-65 correspond to 9.2% 30-days mortality rate while PSI class ≥4 corresponds to 9.3% 30-days mortality rate. However, these scoring systems also have weaknesses. CURB-65 scoring system does not take patient’s comorbidities into account. Therefore, Liu JL et al developed expanded CURB-65 by including LDH, Albumin and platelet count into the scoring system.^9^ Calculating Pneumonia Severity Index class is relatively time-consuming. Moreover, because of the unsatisfactory sensitivity and specificity of these scoring systems, additional risk factors and prognostic markers are needed.^10^

The population is getting older in developed and developing countries. The most important risk factor of community-acquired pneumonia is age. The rate of having community-acquired pneumonia after the age of 65 increases 3-4 times.^11^ The ratio of >65 years-old patients included in our study were 74.6% with 47 patients. This may be caused by comorbid diseases and impaired immune functions in the elderly population. Almost of all the patients who died were in the ≥65 year group.

Thirty five of the patients included in our study had a history of tobacco use and 29 of these patients were male. Smoking substantially increases the risk of pneumonia, invasive pneumococcal disease and Legionella pneumonia. Smoking increases predisposition to bacterial infections by inhibiting the activation of adaptive and innate immune system. This elevated risk can only decrease to similar rates as non-smokers after 10 years of smoking cessation.^12^ Male patients being more than females in the study group are a natural outcome when compared with the literature. In a study by Gutierrez F et al, the incidence of community-acquired pneumonia is 9 cases per 10000 patient-years in females while it is 16 cases in males.^13^

88.8% of the 63 patients included in our study had at least one comorbid disease. In a study of 395 patients by Ruiz M et al., 76% of the patients had a concomitant disease.^14^ The study by Kadakal F et al. found the rate of comorbid diseases to be 23%.^15^ The rate of diseases concomitant with community-acquired pneumonia may be related to the hospital, where the study is conducted. Our study was conducted in a tertiary care hospital and therefore comorbid diseases were higher in number. The most frequent comorbid diseases were hypertension and diabetes mellitus, being present in 57% and 42% of the study patients respectively.

The frequency of Gram negative enteric bacilli and pathogens like *P.aeruginosa* increases in patients with chronic obstructive pulmonary disease.^14^ Similarly, patients with a history of cerebrovascular accident and patient with dementia are more prone to aspiration pneumonia. These comorbid situations these can make the course of community-acquired pneumonia to be more severe.

Bedside qSOFA score, which was defined in The Third International Consensus Definitions for Sepsis and Septic Shock^16^, was also used in our study. There was no significant difference in patient prognosis and endocan levels according to this scoring system which takes altered mental status, low blood pressure and increased respiratory rate into account. qSOFA could not distinguish our patient group.

Mean white blood cell count and C-reactive protein levels were not different between CURB-65 and PSI classes in our study. On the other hand, analyses revealed that endocan levels were significantly lower in groups with CURB-65 score ≥2 and PSI class ≥2 than other groups. Endocan levels have been investigated in pulmonary thromboembolism, detecting acute lung injury after major trauma and sepsis patients; yet there is no study that assesses serum endocan levels in community-acquired pneumonia in the literature. In patient with pulmonary thromboembolism, endocan levels rise as the size of the emboli gets larger.^17^ In contrast, endocan levels are significantly lower in cases that develop acute lung injury after major trauma than cases that don’t develop acute lung injury.^18^

In a study with sepsis patients, serum endocan levels are significantly elevated in patients with severe sepsis and multi-organ failure. Endocan was interpreted as a helpful biomarker in determining the severity and prognosis of sepsis^19^ Scherpereel A et al. reported that endocan levels of patients in septic shock are significantly elevated and mortality rates of patients with high endocan levels are higher.^20^ In our study, endocan levels were not correlated with mortality. The reason behind this may be that the endocan levels increases in sepsis patients while decreases in pneumonia patients. Also, the mortality rate in our study being lower than studies performed in intensive care units may be another cause.

In the literature, endocan levels are increased in clinically more severe patients in pulmonary thromboembolism and sepsis. On the contrary, endocan levels of the patients with higher PSI and CURB-65 scores were lower in the current study. Similarly, Mikkelsen ME et al.^18^ found that patients who develop acute lung injury after trauma have lower endocan levels. Béchard D et al.^21^ showed that endocan, which is a proteglycan, binds LFA-1 that is found on the cell surface of leukocytes and this complex, in turn, decreases leukocyte adhesion through ICAM-1. The cause of relatively more severe patients having lower endocan levels may be neutrophil-derived cathepsin G, which is shown to increase with neutrophil activation.^22^ This protein cleaves endocan, turning it into a 14 kDa peptide fragment.

Serum endocan levels decrease in acute lung injury^18^ as it does in our study with CAP patients. However, studies about sepsis in the literature define elevated endocan levels as a marker of poor prognosis. In the study by Mihajlovicet on sepsis patients, 28% of the patients were in sepsis clinic due to respiratory infections. The majority of these were being treated for pneumonia. Another study with 175 patients^23^, 51.8% of the patients (a total of 73 patients) were in sepsis due to pneumonia. More comprehensive studies on pneumonia patients are warranted for the guidance of endocan levels.

The analysis of our study data revealed that mean endocan levels in the pneumonia group were significantly lower than the control group (p<0.005). However, there was no statistically significant difference when the end-point was death.

Endocan is a good to predict PSI class (AUC: 0.88 and %95 CI 0.80-0.96). When optimal cut-off value for endocan is considered to be 64.96 pg/ml, the sensitivity is 85.2% and the specificity is 83.3%. Similarly, endocan was an adequate parameter to predict CURB-65 scores ≥2 (AUC:0.73 and %95 CI 0.63-0.84). When optimal cut-off value for endocan is considered to be 64.96 pg/ml, the sensitivity is 82.4% and the specificity is 55.6%. According to the current data, when serum endocan level of a pneumonia patient is below 64.96 pg/ml,30-day mortality rate is approximately 9% and hospitalization is recommended.

### Limitations of the study

The limitations of our study are the low number of included patients, high mean age, high rate of comorbid diseases, not being able to measure other biomarkers that indicate neutrophil activation including eosinophilic cationic protein and myeloperoxidase and not measuring endocan levels consecutively or post-treatment. A larger study in a younger population with less comorbidity in which endocan levels are measured consecutively and after treatment is required to determine the role of endocan as a prognostic biomarker in community-acquired pneumonia and its correlation with mortality.

## CONCLUSION

Serum endocan levels in patients with community-acquired pneumonia are significantly lower than control patients. Even though endocan level is not associated with mortality, it indicates the need for inpatient treatment. Endocan levels can increases with systemic inflammatory conditions like sepsis and there can be other confounding factors; therefore patients cannot be evaluated based solely on serum endocan levels, but it can be used as a biomarker to support the clinical opinion of the treating physician.

### Author’s Contribution

**SG & MK** conceived, designed and editing of manuscript.

**UMG** did statistical analysis.

**SG & MK** did data collection and manuscript writing.

**MK** did review and final approval of manuscript.
